# Supplementary material for: The challenge of population aging for mitigating deaths from PM2.5 air pollution in China
Source: Nat Commun. 2023 Aug 26;14:5222. doi: 10.1038/s41467-023-40908-4 (PMC10460422; doi:10.1038/s41467-023-40908-4)
Supplement: Supplementary file 1 — Supplemantrary Material [file 41467_2023_40908_MOESM1_ESM.pdf]

Supplementary materials for

The challenge of population aging for future mitigation of deaths attributable to $PM_{2.5}$ air pollution in China

Fangjin Xu, Qingxu Huang*, Huanbi Yue, Xingyun Feng, Haoran Xu, Chunyang He, Peng Yin, Brett A. Bryan

Contents

Supplementary Table S1 Changes in deaths attributable to $PM_{2.5}$ air pollution in China from 2017 to 2035

Supplementary Table S2 Deaths attributable to $PM_{2.5}$ air pollution among regions in China

Supplementary Table S3 The comparison of predicted deaths attributable to $PM_{2.5}$ air pollution with the GBDMAPS China study

Supplementary Figure S1 Changes in deaths attributable to $PM_{2.5}$ air pollution in China from 2017 to 2035

Supplementary Figure S2 Study area showing the 31 provinces aggregated into North, Northeast, East, Central, South, Southwest, and Northwest regions.

Supplementary Figure S3 The Diseases Considering in This Paper

Supplementary Figure S4 Comparison of deaths attributable to $PM_{2.5}$ air pollution estimates between our results with previous studies

Supplementary Figure S5 Deconstruction analysis diagram

Supplementary Figure S6 Contribution of each driver to deaths attributable to $PM_{2.5}$ air pollution in provinces in China from 2017-2035

Supplementary Figure S7 Deaths attributable to $PM_{2.5}$ air pollution by disease from 2000 to 2035

Supplementary Table S1 Changes in deaths attributable to $PM_{2.5}$ air pollution (DAPP) in China from 2019 to 2035

Province	Change of DAPP (thousand)				Change in DAPP (per 100 km²)			
	SSP1	SSP2	SSP3	SSP5	SSP1	SSP2	SSP3	SSP5
Anhui	-11.92	-0.89	11.69	-3.18	-63.01	-4.36	108.24	-9.58
Beijing	3.47	7.65	7.89	5.25	245.23	427.93	398.84	352.52
Chongqing	-1.07	6.11	8.71	0.37	-4.96	0.33	-200.94	1.25
Fujian	-0.34	4.79	5.93	1.46	-1.54	132.64	66.98	1.7
Gansu	-6.24	-1.71	-2.15	-5.49	-8.84	-2.17	-3.15	-6.81
Guangdong	-3.34	12.49	13.97	2.87	-13.04	59.35	62.46	2.5
Guangxi	-10	-1.26	1.38	-8.39	-23.4	-0.84	-152.75	-16.58
Guizhou	-12.35	-6.21	-3.72	-11.25	-58.96	-34.85	-40.88	-51.91
Hainan	-1.08	-0.03	0.3	-0.81	-78.42	-1.06	9.75	-46.54
Hebei	-24.4	-10.89	-6.19	-21.67	-96.82	-38.88	-22.49	-81.21
Heilongjiang	-0.28	7.75	12.87	1.98	1.14	20.44	28.14	15.44
Henan	-26.21	-8.87	-2.54	-23.69	-116.32	-35.09	-9.05	-100.62
Hubei	-5.55	5.8	10.47	-3.51	-14.34	-23.4	114.3	-6.75
Hunan	-8.76	5.35	10.95	-6.52	-23.64	7.29	-114.41	-15.58
Inner Mongolia	-1.47	3.14	3.49	-0.29	0.55	2.28	2.08	-1.43
Jiangsu	14.52	32.69	34.3	19.88	1512.25	409.58	386.46	499.17
Jiangxi	-9.38	-1.55	0.7	-8.07	-42.33	-6.83	1.02	-34.25
Jilin	0.63	7.55	11.47	2.17	6.82	34.98	48.27	14.6
Liaoning	1.85	12.29	17.07	4.84	2.63	145.12	136.77	-27.68
Ningxia	-2.6	-1.69	-1.63	-2.38	-42.34	-30.1	-30.55	-36.4
Qinghai	-3.36	-2.37	-2.47	-3.02	-3.36	-2.67	-3.71	-2.77
Shaanxi	-6.2	0.14	2.38	-4.58	-20.55	0.4	3.97	-13.44
Shandong	-6.91	14.92	22.09	-2.34	-23.3	-142.76	260.34	-5.49
Shanghai	6.74	11.83	10.79	9.2	1420.76	1950.71	1738.97	1830.31
Shanxi	-10.74	-4.75	-3.07	-9.3	-54.3	-24.71	-20.09	-43.77
Sichuan	-11.55	6.45	12.87	-6.06	-4.08	23.66	34.52	9.96
Tianjin	-1.03	2.21	3.86	-0.99	-60.18	-104.08	352.33	-56.68
Tibet	-1.93	-1.48	-1.56	-1.77	-1.29	-0.89	-1.25	-1.11
Xinjiang	-12.26	-7.47	-8.3	-11.06	-4.31	-2.49	-3.23	-3.23
Yunnan	-16.47	-9.33	-9.79	-14.79	-31.76	-16.53	-16.7	-26.14
Zhejiang	11.32	22.7	21.78	16.05	134.11	209.89	198.96	181.11
Average	-5.06	3.4	5.92	-2.75	-4.09	-6.8	13.26	-1.4

Supplementary Table S2 Deaths attributable to $PM_{2.5}$ air pollution (DAPP) among regions in China

(a) Total DAPP (thousand people)

Year \ Area	North	Northeast	East	Central	South	Southwest	Northwest
SSP1-2.6							
2000	100.99	60.55	227.73	163.23	73.83	129.42	82.42
2005	117.4	77.89	271.03	183.19	104.56	154.87	89.33
2010	127.98	79.18	257.03	181.32	97.88	154.81	93.79
2015	130.84	82.93	237.36	174.11	88.7	144.68	92.05
2020	115.31	96.16	275.52	165.3	91.87	145.77	74.44
2025	107.46	89.72	251.19	148.87	85.02	130.9	71.58
2030	104.35	87.32	254.94	143.1	84.53	127.04	73.68
2035	104.31	85.98	256.67	137.36	85.25	117.31	72.76
SSP2-4.5							
2000	100.99	60.55	227.73	163.23	73.83	129.42	82.42
2005	117.4	77.89	271.03	183.19	104.56	154.87	89.33
2010	127.98	79.18	257.03	181.32	97.88	154.81	93.79
2015	130.84	82.93	237.36	174.11	88.7	144.68	92.05
2020	118.8	99.97	287.64	172.18	96.39	154.33	76.49
2025	119.39	100.6	281.81	166.22	93.94	146.42	77.9
2030	127.04	105.65	310.15	174.86	103.38	158.18	86.21
2035	135.85	111.37	337.13	180.17	110.88	156.22	90.32
SSP3-7.0							
2000	100.99	60.55	227.73	163.23	73.83	129.42	82.42
2005	117.4	77.89	271.03	183.19	104.56	154.87	89.33
2010	127.98	79.18	257.03	181.32	97.88	154.81	93.79
2015	130.84	82.93	237.36	174.11	88.7	144.68	92.05
2020	119.3	99.28	301.92	170.56	96.23	152.46	75.94
2025	122.72	104.87	301.02	171.28	96.78	151.29	78.28
2030	131.63	113.6	326.2	181.26	104.74	157.5	83.56
2035	144.47	125.19	359.93	196.77	115.33	167.2	91.25
SSP5-8.5							
2000	100.99	60.55	227.73	163.23	73.83	129.42	82.42
2005	117.4	77.89	271.03	183.19	104.56	154.87	89.33
2010	127.98	79.18	257.03	181.32	97.88	154.81	93.79
2015	130.84	82.93	237.36	174.11	88.7	144.68	92.05
2020	112.98	94.35	283.22	164.56	90.61	146.87	74.9
2025	106.12	91.17	261.61	149.92	84.8	133.01	71.35
2030	105.96	90.43	274.01	147.87	90.6	133.71	75.46
2035	111.49	92.77	285.62	144.16	93.36	127.18	76.88

(b) DAPP per 100 thousand people

Year \ Area	North	Northeast	East	Central	South	Southwest	Northwest
SSP1-2.6							
2000	76.25	63.39	65.36	83.4	50.24	67.14	103.51
2005	84.4	80.14	75.17	94.81	73.88	84.12	109.9
2010	82.29	79.64	68.01	92.22	65.96	84.11	113.06
2015	78.09	83.98	60.87	86.71	62.55	79.22	106.42
2020	67.26	88.74	64.41	74.86	56.19	69.65	71
2025	63.71	84.89	58.75	67.72	50.26	62.86	67.28
2030	62.8	85.02	60.12	65.87	48.59	61.51	69.36
2035	64.26	87.13	61.26	64.05	47.89	57.94	68.53
SSP2-4.5							
2000	76.25	63.39	65.36	83.4	50.24	67.14	103.51
2005	84.4	80.14	75.17	94.81	73.88	84.12	109.9
2010	82.29	79.64	68.01	92.22	65.96	84.11	113.06
2015	78.09	83.98	60.87	86.71	62.55	79.22	106.42
2020	68.62	91.51	66.6	77.13	58.18	72.75	72.13
2025	69.53	93.91	64.72	74.04	53.82	68.81	71.79
2030	74.81	101.2	71.58	78.39	56.84	74.58	78.61
2035	81.46	110.61	78.54	81.25	58.73	74.51	81.72
SSP3-7.0							
2000	76.25	63.39	65.36	83.4	50.24	67.14	103.51
2005	84.4	80.14	75.17	94.81	73.88	84.12	109.9
2010	82.29	79.64	68.01	92.22	65.96	84.11	113.06
2015	78.09	83.98	60.87	86.71	62.55	79.22	106.42
2020	69.74	90.17	69.48	75.38	56.81	70.89	70.92
2025	71.74	96.64	68.4	74.3	54.63	69	70.74
2030	78.02	107.47	74.48	77.77	56.8	71.08	73.85
2035	87.42	122.14	82.96	83.22	60.07	74.61	79.08
SSP5-8.5							
2000	76.25	63.39	65.36	83.4	50.24	67.14	103.51
2005	84.4	80.14	75.17	94.81	73.88	84.12	109.9
2010	82.29	79.64	68.01	92.22	65.96	84.11	113.06
2015	78.09	83.98	60.87	86.71	62.55	79.22	106.42
2020	65.52	87.21	66.01	74.86	55.43	70.32	71.41
2025	61.77	86.49	60.93	69	50.25	64.43	67.3
2030	62.22	88.52	64.24	69.82	51.57	66.12	71.03
2035	66.49	94.58	67.34	69.72	51.09	64.21	72.63

Supplementary Table S3 The comparison of projected deaths attributable to $PM_{2.5}$ air pollution (DAPP) with the GBDMAPS China study (Group GMW, 2016)

Year	Data resource	Scenarios	DAPP (thousand)
2030	GBDMAPS China	BAU1	1267.4
		BAU2	1144.8
		PC1	1139.2
		PC2	990.1
	This study	SSP1-2.5	875.0
		SSP2-4.5	1065.5
		SSP3-7.0	1098.5
		SSP5-8.5	918.0

BAU1: Business as usual- Twelfth Five-Year Plan for Environmental Protection

BAU2: Business as usual- Maximum Feasible Emission Controls Regardless of Cost

PC1: Air pollution control-Twelfth Five-Year Plan for Environmental Protection

PC2: Air pollution control- Maximum Feasible Emission Controls Regardless of Cost

Supplementary Figure S1 Changes in deaths attributable to $PM_{2.5}$ air pollution (DAPP) in China from 2019 to 2035

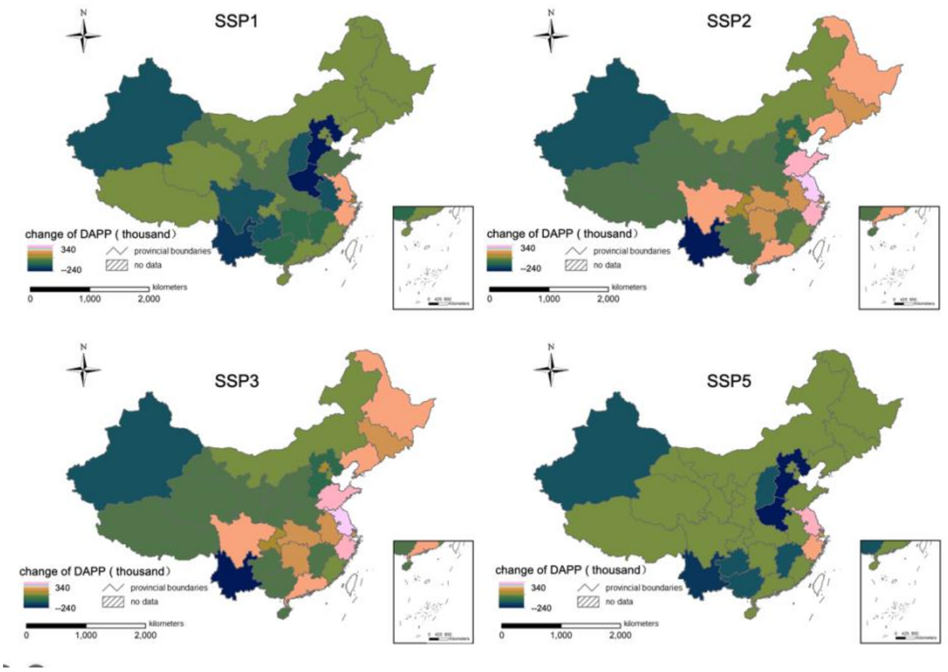

(a. Change in total DAPP in the SSP1 scenario; b. Change in total DAPP in the SSP2 scenario; c. Change in total DAPP in the SSP3 scenario; d. Change in total DAPP in the SSP5 scenario)

Supplementary Figure S2 Study area showing the 31 provinces aggregated into North, Northeast, East, Central, South, Southwest, and Northwest regions.

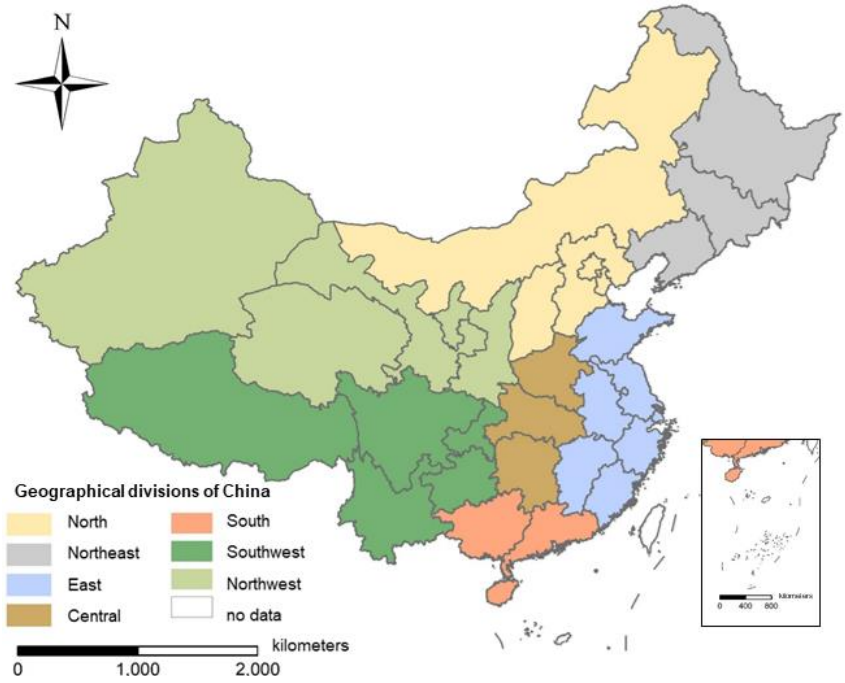

Supplementary Figure S3 The Diseases Considering in This Paper

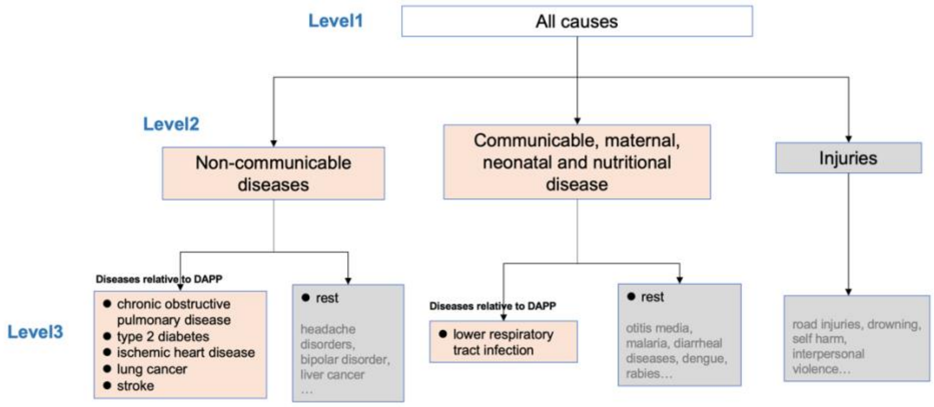

Supplementary Figure S4 Comparison of deaths attributable to $PM_{2.5}$ air pollution (DAPP) estimates between our results with previous studies.

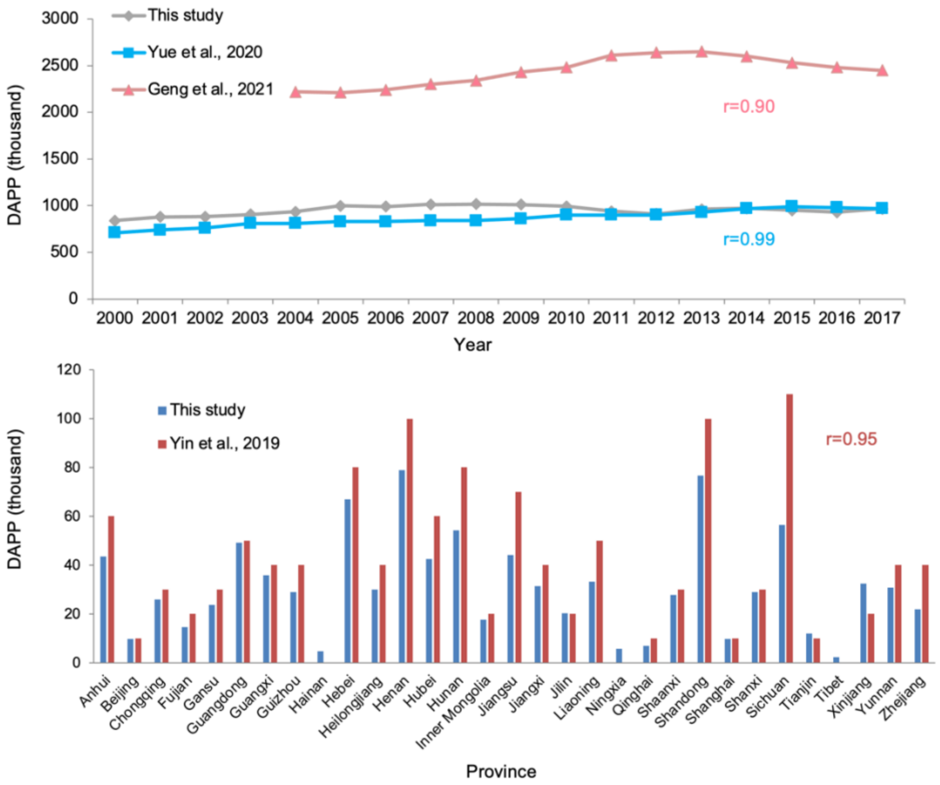

Supplementary Figure S5 Decomposition Analysis Diagram

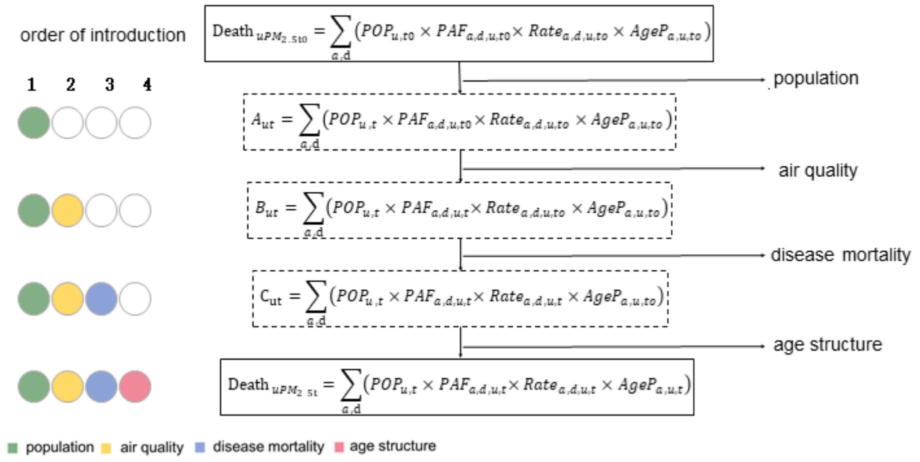

Note: Redrawn from Cohen et al., and 2017 Yue et al., 2020; Note: There are a total of 24 introduction sequences, one of which is in this figure.

Supplementary Figure S6 Contribution of each driver to deaths attributable to $PM_{2.5}$ air pollution (DAPP) in provinces in China from 2019-2035

the proportion of older adults (%)	province	(a) changes of DAPP (thousand)	(a) age structure	(a) $PM_{2.5}$ concentration contribution	(a) population contribution	(a) disease mortality contribution	(b) changes of DAPP (thousand)	(b) age structure	(b) $PM_{2.5}$ concentration contribution	(b) population contribution	(b) disease mortality contribution
22.47	Anhui	-11.92	20.07	-9.38	-3.26	-19.35	-0.89	23.65	-4.25	-2.12	-18.17
26.49	Beijing	3.47	10.89	-3.32	0.84	-4.94	7.65	12.47	-1.34	1.2	-4.67
26.28	Chongqing	-1.07	26.61	-7.19	-3.82	-16.68	6.11	29.16	-4.4	-3.35	-15.31
21.88	Fujian	-0.34	9.79	-4.49	0.19	-5.83	4.79	11.42	-2.17	0.73	-5.2
21.42	Gansu	-6.24	11.07	-2.91	-0.09	-14.3	-1.71	12.43	-1.49	0.65	-13.3
17.91	Guangdong	-3.34	21.78	-12.65	5.23	-17.7	12.49	26.05	-5.9	7.75	-15.41
18.58	Guangxi	-10	18.95	-7.43	1.06	-22.58	-1.26	21.2	-4.07	2.93	-21.32
17.01	Guizhou	-12.35	9.06	-5.95	1.12	-16.58	-6.21	10.34	-3.37	2.53	-15.71
19.81	Hainan	-1.08	3.11	-0.81	0.37	-3.75	-0.03	3.4	-0.52	0.63	-3.55
21.64	Hebei	-24.4	27.66	-12.61	0.49	-39.95	-10.89	31.83	-5.5	2.29	-39.51
31.11	Heilongjiang	-0.28	31.17	-7.32	-2.76	-21.37	7.75	34.9	-3.58	-2.72	-20.84
19.87	Henan	-26.21	34.79	-15.53	-0.1	-45.38	-8.87	39.97	-6.83	2.39	-44.41
25.4	Hubei	-5.55	31.52	-10.48	-1.77	-24.81	5.8	35.65	-5.33	-0.77	-23.75
23.69	Hunan	-8.76	37.65	-12.98	-2.83	-30.6	5.35	42.16	-6.92	-1.21	-28.68
26.1	Inner Mongol	-1.47	13.22	-3.22	-0.51	-10.95	3.14	14.92	-1.18	-0.12	-10.48
27.38	Jiangsu	14.52	51.08	-14.29	-0.35	-21.92	32.69	57.89	-5.92	0.01	-19.3
18.93	Jiangxi	-9.38	12.62	-6.88	-0.44	-14.68	-1.55	14.84	-3.35	0.67	-13.71
31.78	Jilin	0.63	21.55	-5.57	-2.16	-13.19	7.55	24.57	-2.4	-1.51	-13.1
31.8	Liaoning	1.85	35.04	-9.46	-3.3	-20.43	12.29	39.34	-3.7	-3.38	-19.98
16.63	Ningxia	-2.6	0.67	-0.73	0.28	-2.82	-1.69	0.87	-0.34	0.48	-2.71
18.25	Qinghai	-3.36	1.49	-0.5	0.04	-4.39	-2.37	1.76	-0.22	0.27	-4.18
23.47	Shaanxi	-6.2	17.09	-5.99	-1.12	-16.19	0.14	19.16	-3.06	-0.55	-15.4
25.94	Shandong	-6.91	59.81	-19.09	-4.13	-43.5	14.92	67.62	-8.62	-2.43	-41.65
29.92	Shanghai	6.74	15.66	-3.46	-0.93	-4.53	11.83	17.75	-1.34	-0.68	-3.89
21.67	Shanxi	-10.74	11.97	-5.52	-0.46	-16.73	-4.75	13.82	-2.52	0.21	-16.27
25.6	Sichuan	-11.55	54.29	-17.68	-7.45	-40.71	6.45	60.68	-9.03	-6.64	-38.56
25.52	Tianjin	-1.03	9.34	-2.66	-2.04	-5.67	2.21	10.63	-1.06	-1.89	-5.46
13.5	Tibet	-1.93	-0.02	-0.2	-0.18	-1.54	-1.48	0.07	0.1	-0.12	-1.53
17.11	Xinjiang	-12.26	9.36	-1.99	0.66	-20.29	-7.47	10.58	-0.97	1.96	-19.04
17.57	Yunnan	-16.47	6.84	-4.69	0.2	-18.81	-9.33	8.51	-0.88	1.33	-18.28
26.72	Zhejiang	11.32	26.86	-8.38	3.7	-10.86	22.7	31.05	-3.78	4.83	-9.41

(a) SSP1-2.6 (b) SSP2-4.5

the proportion of older adults (%)	province	(c) changes of DAPP (thousand)	(c) age structure	(c) $PM_{2.5}$ concentration contribution	(c) population contribution	(c) disease mortality contribution	(d) changes of DAPP (thousand)	(d) age structure	(d) $PM_{2.5}$ concentration contribution	(d) population contribution	(d) disease mortality contribution
22.47	Anhui	11.69	29.29	1.6	1.06	-20.25	-3.18	33.71	-8.43	-5.79	-22.68
26.49	Beijing	7.89	11.36	0.48	-0.04	-3.91	5.25	10.81	-2.4	2	-5.17
26.28	Chongqing	8.71	25.79	0.61	-1.81	-15.88	0.37	27.34	-5.47	-4.6	-16.9
21.88	Fujian	5.93	9.65	0.4	0.79	-4.91	1.46	10.01	-3.05	0.55	-6.05
21.42	Gansu	-2.15	9.33	-0.22	1.76	-13.01	-5.49	11.42	-1.97	-0.5	-14.45
17.91	Guangdong	13.97	20.84	1.49	5.85	-14.21	2.87	21.7	-8.85	8.49	-18.47
18.58	Guangxi	1.38	16.03	0.79	5.68	-21.13	-8.39	19.81	-5.56	0.14	-22.77
17.01	Guizhou	-3.72	6.57	0.6	4.8	-15.7	-11.25	9.71	-4.51	0.29	-16.75
19.81	Hainan	0.3	2.81	0.12	0.83	-3.45	-0.81	3.15	-0.57	0.41	-3.8
21.64	Hebei	-6.19	25.05	1.64	4.52	-37.4	-21.67	28.27	-9.79	0.05	-40.19
31.11	Heilongjiang	12.87	33.2	1.11	-1.71	-19.74	1.98	32.22	-5.07	-3.33	-21.84
19.87	Henan	-2.54	30.98	1.53	7.28	-42.33	-23.69	36.09	-11.87	-2.23	-45.68
25.4	Hubei	10.47	30.64	0.95	2.01	-23.13	-3.51	32.76	-7.88	-3.2	-25.19
23.69	Hunan	10.95	35.61	1.24	2.88	-28.79	-6.52	39.15	-9.87	-4.86	-30.94
26.1	Inner Mongol	3.49	12.84	0.03	0.22	-9.59	-0.29	13.5	-2.19	-0.46	-11.15
27.38	Jiangsu	34.3	50.83	2.36	0.44	-19.33	19.88	51.4	-10.05	0.98	-22.45
18.93	Jiangxi	0.7	10.92	0.7	2.63	-13.55	-8.07	13.35	-5.02	-1.52	-14.88
31.78	Jilin	11.47	23.49	1.02	-1.01	-12.03	2.17	22.19	-3.96	-2.55	-13.51
31.8	Liaoning	17.07	37.38	1.64	-3.51	-18.44	4.84	35.63	-6.68	-3.15	-20.96
16.63	Ningxia	-1.63	0.31	-0.01	0.61	-2.54	-2.38	0.69	-0.53	0.31	-2.85
18.25	Qinghai	-2.47	1.2	-0.04	0.46	-4.1	-3.02	1.55	-0.17	0.06	-4.47
23.47	Shaanxi	2.38	16.52	0.2	0.37	-14.71	-4.58	17.48	-4.26	-1.42	-16.38
25.94	Shandong	22.09	59.54	2.91	-0.12	-40.23	-2.34	60.77	-14.58	-4.53	-44
29.92	Shanghai	10.79	16.27	0.58	-2.4	-3.65	9.2	15.59	-2.26	0.63	-4.76
21.67	Shanxi	-3.07	11.07	0.33	1.04	-15.51	-9.3	12.25	-4.11	-0.6	-16.85
25.6	Sichuan	12.87	54.14	1.27	-2.83	-39.71	-6.06	56.84	-11.41	-9.76	-41.73
25.52	Tianjin	3.86	11.11	0.44	-2.53	-5.16	-0.99	7.87	-1.96	-1.4	-5.49
13.5	Tibet	-1.56	-0.08	0.02	-0.07	-1.44	-1.77	0	-0.01	-0.17	-1.6
17.11	Xinjiang	-8.3	7.38	-0.07	2.62	-18.23	-11.06	9.26	-1.1	1	-20.22
17.57	Yunnan	-9.79	4.9	0.55	2.43	-17.67	-14.79	7.26	-2.94	0.05	-19.15
26.72	Zhejiang	21.78	27.32	1.1	2.61	-9.24	16.05	26.81	-5.51	6.1	-11.35

(c) SSP3-7.0 (d) SSP5-8.5

Supplementary Figure S7 Deaths attributable to $PM_{2.5}$ air pollution (DAPP) by disease from 2000 to 2035

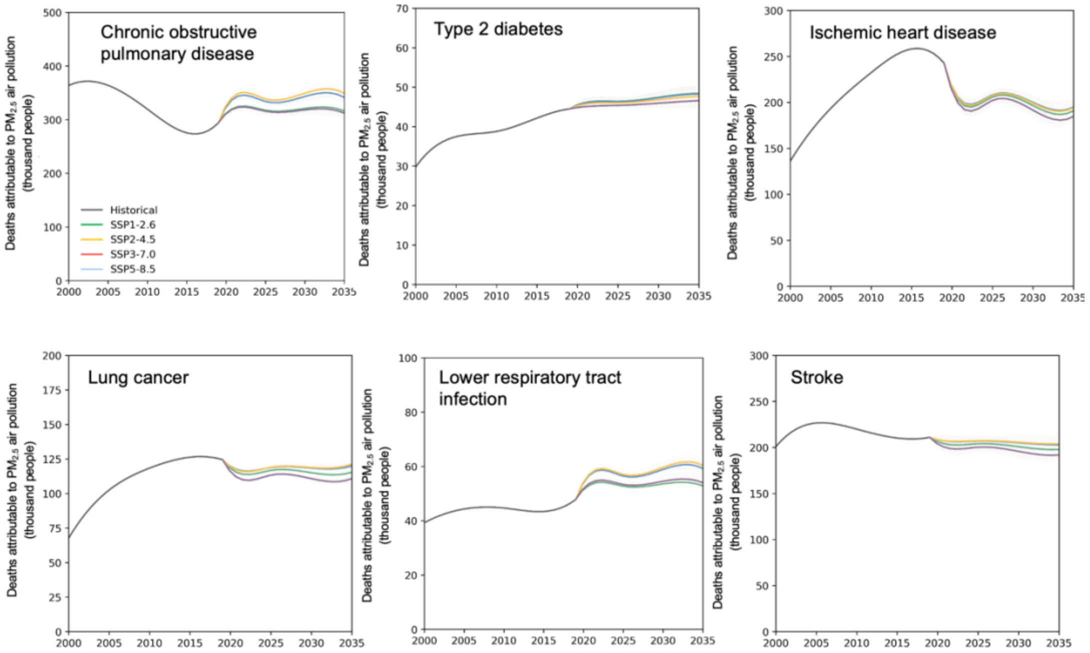

Note: COPD, DM2, IHD, LC, and LRI refer to chronic obstructive pulmonary disease, type 2 diabetes, ischemic heart disease, lung cancer, and lower respiratory tract infection, respectively. Data are presented as mean values, and shading indicates the 95% confidence interval.

References

Apte, J. S., Marshall, J. D., Cohen, A. J. & Brauer, M. Addressing Global Mortality from Ambient PM2.5. Environmental Science & Technology 49, 8057-8066, doi:10.1021/acs.est.5b01236 (2015)

Feng D A , Jla B , Xz A , et al. Decoupling relationship between haze pollution and economic growth: A new decoupling index[J].2021. Ecological Indicators, 129.

FengDong, BolinYu, YulingPan. Examining the synergistic effect of CO2 emissions on PM2.5 emissions reduction: Evidence from China[J]. Journal of Cleaner Production, 2019.

Group GMW. Burden of disease attributable to coal-burning and other air pollution sources in China. Special report 2016; 20.

Yue, H., He, C., Huang, Q., Yin, D. & Bryan, B. A. Stronger policy required to substantially reduce deaths from PM2.5 pollution in China. Nature Communications 11, doi:10.1038/s41467-020-15319-4 (2020).

Zha D , Liu P , Shi H . Does population aging aggravate air pollution in China?[J]. Mitigation and Adaptation Strategies for Global Change, 2022, 27(2).
