# Supplementary material for: The challenge of population aging for mitigating deaths from PM2.5 air pollution in China
Source: Nat Commun. 2023 Aug 26;14:5222. doi: 10.1038/s41467-023-40908-4 (PMC10460422; doi:10.1038/s41467-023-40908-4)
Supplement: Supplementary file 3 — Reporting Summary [file 41467_2023_40908_MOESM3_ESM.pdf]

nature portfolio

Corresponding author(s): Qingxu Huang

Last updated by author(s): Jul 9, 2023

Reporting Summary

Nature Portfolio wishes to improve the reproducibility of the work that we publish. This form provides structure for consistency and transparency in reporting. For further information on Nature Portfolio policies, see our Editorial Policies and the Editorial Policy Checklist.

Statistics

For all statistical analyses, confirm that the following items are present in the figure legend, table legend, main text, or Methods section.

n/a	Confirmed	
☐	☒	The exact sample size (*n*) for each experimental group/condition, given as a discrete number and unit of measurement
☒	☐	A statement on whether measurements were taken from distinct samples or whether the same sample was measured repeatedly
☒	☐	The statistical test(s) used AND whether they are one- or two-sided<br>*Only common tests should be described solely by name; describe more complex techniques in the Methods section.*
☒	☐	A description of all covariates tested
☒	☐	A description of any assumptions or corrections, such as tests of normality and adjustment for multiple comparisons
☐	☒	A full description of the statistical parameters including central tendency (e.g. means) or other basic estimates (e.g. regression coefficient) AND variation (e.g. standard deviation) or associated estimates of uncertainty (e.g. confidence intervals)
☒	☐	For null hypothesis testing, the test statistic (e.g. *F*, *t*, *r*) with confidence intervals, effect sizes, degrees of freedom and *P* value noted<br>*Give P values as exact values whenever suitable.*
☒	☐	For Bayesian analysis, information on the choice of priors and Markov chain Monte Carlo settings
☒	☐	For hierarchical and complex designs, identification of the appropriate level for tests and full reporting of outcomes
☐	☒	Estimates of effect sizes (e.g. Cohen's *d*, Pearson's *r*), indicating how they were calculated

Our web collection on statistics for biologists contains articles on many of the points above.

Software and code

Policy information about availability of computer code

Data collection	No code or software was used in data collection.
Data analysis	We used the integrated exposure-response function updated in the Global Burden of Disease 2019 to estimate the relative risk caused by PM2.5 exposure. The detailed function is accessible to all users at https://doi.org/10.6069/630D-5V32. Custom Python (3.8.3) scripts for estimating disease mortality and the functions used for estimating the deaths attributable to PM2.5 air pollution embedded in the Microsoft Excel file were available at Github (doi: 10.5281/zenodo.8128795). Other data processing and map creation in this study are conducted at ArcGIS (10.6) and Microsoft Excel (16.74).

For manuscripts utilizing custom algorithms or software that are central to the research but not yet described in published literature, software must be made available to editors and reviewers. We strongly encourage code deposition in a community repository (e.g. GitHub). See the Nature Portfolio guidelines for submitting code & software for further information.

Data

Policy information about availability of data

All manuscripts must include a data availability statement. This statement should provide the following information, where applicable:

- Accession codes, unique identifiers, or web links for publicly available datasets
- A description of any restrictions on data availability
- For clinical datasets or third party data, please ensure that the statement adheres to our policy

All the data created in this study are openly available at Github repositories with the identifier https://github.com/q22huang/DAPAP. The source data underlying

Figs. 1–5 are provided as a Source Data file. Historical data of concentration of PM2.5 on surface from Global PM2.5 Assessment Dataset can be obtained at http://fizz.phys.dal.ca/~atmos/. The disease mortality data (national scale) from GBD2019 can be obtained at https://vizhub.healthdata.org/gbd-results/. The historical data of population, GDP per capita, proportion of secondary industry, educational level, fertility rate (provincial scale) can be obtained from China Statistical Yearbooks. The projected data of Sea salt, sulfate, organic aerosol, black carbon (spatial resolution 1°) from CMIP6 dataset can be obtained at https://pcmdi.llnl.gov/CMIP6/. The projected data of population datasets in China from Chen et al can be obtained at https://doi.org/10.6084/m9.figshare.c.4605713.v1.

Human research participants

Policy information about studies involving human research participants and Sex and Gender in Research.

Reporting on sex and gender	This study doesn't involve sex or gender.
Population characteristics	This is not applicable in this study.
Recruitment	This is not applicable in this study.
Ethics oversight	This is not applicable in this study.

Note that full information on the approval of the study protocol must also be provided in the manuscript.

Field-specific reporting

Please select the one below that is the best fit for your research. If you are not sure, read the appropriate sections before making your selection.

☐ Life sciences ☐ Behavioural & social sciences ☒ Ecological, evolutionary & environmental sciences

For a reference copy of the document with all sections, see nature.com/documents/nr-reporting-summary-flat.pdf

Ecological, evolutionary & environmental sciences study design

All studies must disclose on these points even when the disclosure is negative.

Study description	We estimated the annual deaths attributable to ambient PM2.5 pollution in China from 2020 to 2035 at a pixel scale (10km) by combining an epidemiological model and climate scenarios.
Research sample	We used age- and disease specific death rate data from Global Burden of Disease 2019 (https://vizhub.healthdata.org/gbd-results/). Six kinds of diseases related to PM2.5 pollution were considered in this study, including lung cancer, chronic obstructive pulmonary disease, lower respiratory infection, ischemic heart disease, stroke, and diabetes mellitus type 2. We chose them because they are the major contributors to the deaths attributable to ambient PM2.5 pollution in China. Fifteen age groups were included in the equation, i.e., 25–30, 30–35...90–95, and beyond 95 years old. For lower respiratory infection, children less than 5 years old were also considered. Such 5-year age group data are the most up-to-date data for estimating the deaths attributable to ambient PM2.5 pollution in China.
Sampling strategy	The 5-year age group and the 6 selected diseases were chosen following the Global Burden of Disease 2019 study (https://vizhub.healthdata.org/gbd-results/). Such strategy is widely adopted in related studies.
Data collection	The PM2.5 data, demographic data and death rate data were download directly by Fangjin Xu and Huanbi Yue. Detailed links were given as below. Historical data of concentration of PM2.5 on surface from Global PM2.5 Assessment Dataset can be obtained at http://fizz.phys.dal.ca/~atmos/. The disease mortality data (national scale) from GBD2019 can be obtained at https://vizhub.healthdata.org/gbd-results/. The historical data of population, GDP per capita, proportion of secondary industry, educational level, fertility rate (provincial scale) can be obtained from China Statistical Yearbooks. The projected data of Sea salt, sulfate, organic aerosol, black carbon (spatial resolution 1°) from CMIP6 dataset can be obtained at https://pcmdi.llnl.gov/CMIP6/. The projected data of population datasets in China from Chen et al can be obtained at https://doi.org/10.6084/m9.figshare.c.4605713.v1.
Timing and spatial scale	The data used in this study were collected between January 2022 to April 2023. The PM2.5 data have a resolution of 0.01 degrees. Population distribution data have a spatial resolution of 0.083 degrees. Data on the age structure of China's population, as well as the age- and disease-specific death rates were obtained from the Global Burden of Disease 2019 dataset, at a provincial and national scale. During the historical period, all these data a available annually from 2000 to 2019, the future projection include data in 2020 and 2035.
Data exclusions	No data were excluded in this study.
Reproducibility	Three attempts to repeat the results, which were independently conducted by our co-authors, were successful.
Randomization	N/A. This study does not involve group allocation and sample randomization.
Blinding	N/A. This study does not involve group allocation and investigator blinding.

Did the study involve field work? ☐ Yes ☒ No

Reporting for specific materials, systems and methods

We require information from authors about some types of materials, experimental systems and methods used in many studies. Here, indicate whether each material, system or method listed is relevant to your study. If you are not sure if a list item applies to your research, read the appropriate section before selecting a response.

Materials & experimental systems

n/a	Involved in the study	
☒	☐	Antibodies
☒	☐	Eukaryotic cell lines
☒	☐	Palaeontology and archaeology
☒	☐	Animals and other organisms
☒	☐	Clinical data
☒	☐	Dual use research of concern

Methods

n/a	Involved in the study	
☒	☐	ChIP-seq
☒	☐	Flow cytometry
☒	☐	MRI-based neuroimaging
